# Prevalence of Metabolic Syndrome in Children and Adolescents with Type 1 Diabetes Mellitus and Possibilities of Prevention and Treatment: A Systematic Review

**DOI:** 10.3390/nu13061782

**Published:** 2021-05-23

**Authors:** Monika Grabia, Renata Markiewicz-Żukowska, Katarzyna Socha

**Affiliations:** Department of Bromatology, Faculty of Pharmacy with the Division of Laboratory Medicine, Medical University of Białystok, Mickiewicza 2D Street, 15-222 Białystok, Poland; monika.grabia@umb.edu.pl (M.G.); katarzyna.socha@umb.edu.pl (K.S.)

**Keywords:** type 1 diabetes mellitus, pediatric diabetes, metabolic syndrome, obesity, children, adolescents, nutrition, physical activity, lifestyle, microbiome

## Abstract

Overweight and obesity are an increasingly common problem, not only among the healthy population, but also in adolescents with type 1 diabetes (T1DM). Excess body weight is related to many cardiometabolic complications as well as a high risk of metabolic syndrome (MetS). The purpose of this systematic review is to provide a concise and critical overview of the prevalence of MetS in children and adolescents with T1DM and, ultimately, to discuss prevention and treatment options. The study was conducted in accordance with PRISMA guidelines. This review shows that, apart from the growing percentage of overweight and obese children and adolescents with T1DM (on average 20.1% and 9.5%, respectively), the problem of the increasing incidence of MetS (range from 3.2 to 29.9%, depending on the criteria used) is one of the most important phenomena of our time. One of the methods of prevention and treatment is a combined approach: changing eating habits and lifestyle, but there are also reports about the beneficial effects of the gut microflora.

## 1. Introduction

Type 1 diabetes mellitus (T1DM) is an autoimmune disease where islet β cells are degraded by the body’s immune system. These cells are responsible for the secretion of insulin; their death means that less and less of this hormone is released [1]. For many years, the incidence of T1DM has been increasing by about 130,000 new cases each year. Currently, according to a report by the International Diabetes Federation (IDF), T1DM affects over 1.1 million children and adolescents under the age of 20 worldwide [2]. Unfortunately, the rates of overweight and obesity are steadily rising, not only among the healthy population, but also in adolescents with T1DM [3]. Excessive body weight is associated with a heightened risk of cardiometabolic complications [4]. Metabolic syndrome (MetS) is defined as a set of multiple factors (physiological, biochemical and metabolic ones) which directly increase the risk of atherosclerotic cardiovascular diseases (CVD). These are a complication that should be prevented in people with diabetes mellitus (DM) [5]. In 2007, a group of experts from around the world gathered to develop a consensus on the definition of MetS [6]. The IDF recommended that the criteria for patients above 16 years old should be similar to those applied to the adult population, but for children and adolescents between 10 and 16 years of age, they should be adjusted for percentile grids. MetS cannot be diagnosed under 10 years of age unless there are disturbances in these parameters in the family history. In 2009, the American Heart Association published its statement in which it recommended additional identification of cardiometabolic risk but did not specify the exact definition of MetS for the pediatric population [7].

The above-mentioned aspects emphasize the importance of early diagnosis of children in order to prevent the increased risk of comorbid cardiometabolic diseases. An international consensus on the criteria should be reached so that preventive examination can be performed before the syndrome becomes manifest. The incidence of MetS is widely reported in adults, including DM patients [8]. However, over the past decade, attention has been drawn to the fact that certain components of MetS have begun to appear in children with T1DM.

The purpose of this systematic review is to provide a concise and critical overview of the prevalence of MetS in children and adolescents with T1DM and discuss the possibilities of prevention and treatment.

## 2. Materials and Methods

### 2.1. Search Strategy and Selection Criteria

**Figure 1:**
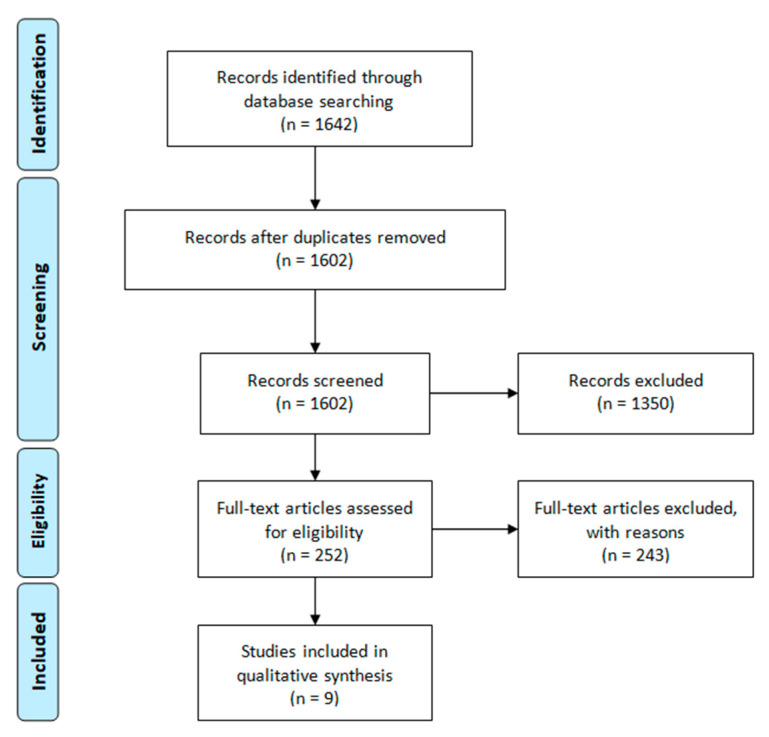
Flow diagram for systematic review.

Our systematic review follows the PRISMA guidelines [9]. A search was conducted on PubMed, Scopus, Web of Science and Cochrane Library in May 2020 and updated for studies published up to October 2020. The MeSH terms are shown in Table S1. Analogous terms were used to search other databases. The following were exclusion criteria from this review: adult age (above 21 years of age), non-T1DM, non-English language, animal/cell studies and case-reports. The investigation involved describing the occurrence of MetS using guidelines (Table 1) proposed by, e.g., IDF [6], Adult Treatment Panel III (ATP) [10], Weiss et al. [11], World Health Organization (WHO) [12] in pediatric patients (up to 21 years of age) diagnosed with T1DM (e.g., presence of anti-GAD (Glutamic Acid Decarboxylase) or anti-insulin antibodies). Two reviewers assessed the studies based on the selection criteria and all divergences were resolved by consensus. The search strategy using the PRISMA scheme is shown in Figure 1.

### 2.2. Data Extraction and Assessment of Study Quality

Baseline characteristics (study design, author, year), study cohorts (number of participants, age, country, definition of MetS, components of MetS) and outcomes were extracted into an MS Excel worksheet. Included studies were assessed using the “Quality Assessment Tool for Observational Cohort and Cross-Sectional Studies” [13]. The questionnaire consists of 14 questions regarding group representativeness, study group recruitment, appropriately selected diagnostic criteria, complications and possible outcome bias. Each question could be answered with “yes” (the item scored 1 point), “no” or “other” (c/d, cannot determine; n/a, not applicable; n/r, not reported). After counting the scores, every study was placed into a quality category (good, fair or poor). Only studies of good or fair quality (for cross-sectional (C-S) studies—from 6 and 4 points, respectively) were included in the review. All divergences of assessment were resolved by consensus.

## 3. Results

### 3.1. Identification of Studies

The search yielded 1642 citations (Figure 1). All of them were screened and 40 duplicate articles were removed. Using the selection criteria, 1602 papers were reviewed by title and abstract and 1350 were excluded. A further 252 studies were identified for full-text assessment and 243 papers were excluded. Nine articles qualified for the quality evaluation. All of them were positively assessed and included in this systematic review [14,15,16,17,18,19,20,21,22]. The most common reasons for rejection were diabetes mellitus other than type 1, adult population or a mixed population of adults and children without the possibility of isolating data for the pediatric patients, and lack of both percentage and numerical data presenting the incidence of MetS in the cohorts.

### 3.2. Study Characteristics

The characteristics of the included studies are summarized in Table 2. All the papers are C-S studies. As regards T1DM diagnosis, five articles are consistent with the American Diabetes Association (ADA) criteria [1] and participants had a marked presence of anti-GAD or anti-insulin antibodies [14,16,17,18,21]. However, in the remaining four research projects, patients also came from hospital clinics [15,19,20,22]. There was no conflict of interest in any of the studies. Most of them (*n* = 7) used the IDF definition [14,16,17,19,20,22]; one used criteria consistent with the ATP [18] and Weiss et al. [21], and another used the IDF, ATP and WHO guidelines [15]. Six studies were conducted in Europe [14,15,16,20,21,22], two in Asia [17,18] and one in Africa [19].

### 3.3. Methodological Quality

The assessment of methodological quality of the included studies is shown in Supplementary Materials Table S2. In accordance with the interpretative recommendations, the research assessment scale was modified for the evaluation of C-S studies. As recommended by the National Institutes of Health [13], for questions no. 6 and 7, all papers were rated “no” and for question no. 8—“n/a”. In the first two cases, this was due to the fact that in C-S studies, exposure and its outcomes are measured over the same time interval. The rationale for the third case is that only two exposures were possible in the study (“yes” or “no”), but this should not negatively affect the final quality assessment. Each of the two researchers separately rated each of the papers according to a modified scale, and in case of discrepancies, the grades were awarded after mutual consensus. Of the nine articles, 5 were of good and 4 of fair quality.

### 3.4. Prevalence of Metabolic Syndrome

The number of people in all the studies included in the review is 1847, the gender distribution is equal—923 boys and 924 girls. As two publications by the same author described the same cohort, only one group was included in the above estimate in order to avoid artificially increasing the size of the sample [17,18]. The study which was the only one involving a population of girls (*n* = 42), conducted by Castro-Correia et al. [14], was the smallest. Among the studies conducted in both sexes, the ones by Saki et al. [17,18] had the smallest cohort (*n* = 87). It was tested by two different criteria of MetS in two publications. The largest sample size (*n* = 500) was found in the study by Łuczyński et al. [16], and the narrowest age groups in the papers by Valerio et al. (16–19 years) [22] and Castro-Correia et al. (14–18 years old) [14]. It was very difficult to estimate the incidence of MetS in studied cohorts due to the high number of different criteria that had been used. The overall percentage of MetS in the studied populations ranged from 3.2% to 29.9%, depending on the criteria selected by the authors. MetS was observed in 90 girls (76 according to the IDF criterion, 14 according to the ATP) and in 52 boys (40 and 12, respectively). Fasting glucose levels were measured in only one cohort [17,18]. The problem of obesity was experienced on average by 9.5% (range: 3.9–14.6%) of the subjects, and 20.1% (range: 9.5–29.3%) were overweight. The average HbA1c value was 8.5% (range: 8.0–9.4%). Abdominal obesity was found in 232 people (14.7% of the entire study population that took the criterion into account), low high-density lipoprotein (HDL) levels in 234 (14.9%), high triglycerides and blood pressure in 201 (12.8%) and 224 (12.6%), respectively.

.

## 4. Discussion

### 4.1. Problem of Overweight, Obesity and Metabolic Syndrome in Healthy and Diabetes Population

The main problem among people with MetS is overweight and obesity. In our review, both of these conditions occurred on average in 20.1% (range: 9.5–29.3%) and 9.5% (range: 3.9–14.6%) of the studied populations, respectively. This may be due to many factors: research of the International SWEET Registry, collecting data from nearly 60 diabetes centers, highlights that there is a significant correlation with age, duration of DM and metabolic control (HbA1c) [23]. Moreover, the problem is also more common among healthy children because—according to WHO data from 2016—over 340 mln children between 5 and 19 years of age have been diagnosed as overweight or obese [24]. It was found that the highest BMI Z-scores occurred in adolescents with T1DM between 15 and 20 years of age [25]. According to the SWEET Registry, every 5 years this indicator increases on average by 0.5 [23]. One explanation of this phenomenon may be excessive consumption of high-energy food (in the form of, e.g., liquid glucose, sweet beverages or candy) for fear of hypoglycemia. Moreover, insulin is an anabolic hormone and, if secreted in excess, can stimulate the appetite, which in the long term may result in weight gain. Thus, the longer the duration of DM, poor metabolic control (high HbA1c) and high insulin resistance, the greater the predisposition to overweight or obesity [25,26].

The percentage of MetS in the presented set of studies ranges from 3.2% to 29.9%, depending on the criteria used, but in overweight and obese children and adolescents without DM, it ranges from 2.8% to 29.3% and from 10% to 66%, respectively [27]. Despite the considerable discrepancy in our results, it should be noted that this is still a quite large percentage. Some studies have a fairly wide age range of the cohort, which can be an advantage because it means an extensive range of patients in whom MetS can be diagnosed. However, on the other hand, this can also distort the results as, according to the IDF criteria, MetS should not be diagnosed in children under 10 years of age. This highlights the first problem related to evaluating the diagnosis in children. Another problem encountered by the researchers is the fasting glucose level criterion. Some authors have concluded that since T1DM is characterized by fluctuations in glycemia resulting from the pathophysiology of the disease, the criterion should be considered as met and one group of researchers tested each person for fasting glucose levels.

The prevalence of MetS in the adult population with T1DM is 8–45%, depending on age and definition [8]. The incidence rate is higher in the elderly and when using the WHO criteria. This is probably because of the inclusion of microalbuminuria as a component of MetS [8,28]. Despite strictly defined criteria in the adult population, in children, there are no homogeneous and objective criteria for the diagnosis of MetS. The development of standards should be based on the outcomes related to appropriate norms for sex, age and ethnicity, such as those proposed by Weiss et al. [11]. Taking into consideration the Polish national percentile grids [29] as an example, it was proposed to diagnose central obesity above the 95th percentile. This value does not coincide with the IDF cut-off point (90th percentile). The suggested cut-off points for hypertension are based on a specific numerical value (≥130/85 mmHg), and this value may be normal for tall boys [30]. A similar issue was discussed by Ahrens et al. [31], who emphasized that high BP cut-off values could contribute to the low percentage of children classified as having MetS. Ferranti et al. [32] compared the values of lipid parameters to their grids and observed that the level of HDL (40 mg/dL) was 10–25th percentile for boys, and the 10–15th percentile for girls, which is lower than the 40th percentile for adults. A similar phenomenon occurs in the case of the cut-off points for triglycerides (110 mg/dL), which are higher than in the adult population (85–95th vs. 75–85th percentile). Each MetS parameter is related to a number of health complications. Some writers have suggested that MetS is associated with an increased risk of diabetes complications (nephropathy, neuropathy and retinopathy) [28,33,34,35]. Obesity is associated with an increased need for insulin and worse metabolic control, which increases the chances of developing atherosclerotic complications and possible hospitalization due to CVD [4,36]. In addition, as an individual factor, it exacerbates the risk of orthopedic complications, cholecystitis and the appearance of psychosocial symptoms in children [37]. Adolescents with T1DM are predisposed to cardiometabolic complications [38], often regardless of body weight but, worryingly, their development of obesity can sometimes result in the development of “double diabetes” with type 2 diabetes mellitus (T2DM). Pozzilli et al. described several such case studies [39]. Some studies indicate an association of low HDL cholesterol with deteriorated metabolic control, which increases the likelihood of micro and macrovascular complications [40]. People with higher levels of this cholesterol fraction are less likely to develop neuropathy [41]. The occurrence of hypertension is associated with diabetic nephropathy, weight gain and insulin resistance [42].

Each of these factors individually significantly increases the risk of health complications, and their simultaneous occurrence may additionally accelerate and intensify them. Therefore, it is important not to focus only on analyzing individual parameters, but to identify all MetS components simultaneously.

Cluster tracking studies have found that some cardiovascular risk factors may persist into later life [43]. Due to the growing statistics of obesity and comorbidities in children, screening of metabolic risk groups is of great importance for the primary prevention of atherosclerosis [44].

Recent years of above research have shown that obesity-related complications have become a very common phenomenon in the pediatric population, which underlines the urgent need to create a new definition of MetS to assist in the early diagnosis and prevention of cardiometabolic disorders. It would be important to develop additional and better MetS markers to enrich diagnostics, thus helping clinicians recognize the warning signs in time.

### 4.2. Strategies for the Prevention and Treatment of Metabolic Syndrome

#### 4.2.1. Diet

The main goal of preventing obesity among children is to promote a healthy lifestyle through a balanced diet (increased consumption of fruit and vegetables, avoiding sweetened drinks, refined carbohydrates and processed foods), appropriate health habits (screen and sleep time) and physical activity [45]. Kamath et al. conducted a meta-analysis which showed a small but statistically significant positive effect of lifestyle interventions on the reduction of unhealthy habits (−0.15; CI = −0.22 to −0.08) and sedentary behavior (−0.29; CI = −0.35 to −0.22) [46]. Excessive consumption of calories that come from fat and low intake of fiber, fruit and vegetables has been associated with the risk of CVD in people with T1DM [47,48]. A proper diet reduces facilitates weight control [49] and is correlated with better glycemic control [50] and prevention of cardiometabolic diseases in adolescents with T1DM [47].

Research highlights that healthy eating patterns, such as the Mediterranean diet (MD) and the Dietary Approaches to Stop Hypertension (DASH) diet have a positive effect on improving the parameters of people with MetS. A large pro-health role is attributed to increased consumption of fish, whole grains, vegetables, legumes and dairy products, but also of such nutrients as: antioxidants, calcium and B vitamins. Many studies emphasize the importance of curbing the consumption of red meat, simple carbohydrates and products with a high glycemic index and glycemic load [51].

It has been shown that a very helpful way to reduce the occurrence of MetS in children was to implement the MD, which is characterized by high consumption of olive oil, vegetables and grains, and reduced consumption of red meat and sweets. There was an 11% decrease in the incidence of MetS (16% up to 5%) in people on the MD compared to control group [52]. Other studies have found a 2.5-fold increased risk of MetS from consuming highly processed foods and more than a 5-fold increased risk from consuming sugar-sweetened beverages (SSB) [53,54]. Unfortunately, no studies have been conducted to assess the effect of excluding SSB on MetS, but one study has revealed that their elimination may improve body weight [55]. Therefore, MetS patients should strive to reduce the amount of SSB, saturated fats and highly processed foods in their diets and consume more oils and vegetables.

Asghari et al. conducted a study on 425 healthy children (6–18 years old) with MetS and found that following the DASH diet resulted in a 64% lower risk of MetS and, along with higher scores on this diet, correlated with lower BP, fasting glucose level and abdominal obesity [56]. A study by Peairs et al. investigated the DASH diet and its modified version (30% of calories from fat, 50% from carbohydrates, 20% from protein) adapted to the young with T1DM and compliant with the ADA guidelines. It was shown that the modified version resulted in reducing the levels of glucose, and thus better glycemic control and a reduction in the number of hyperglycemic incidents. In addition, the quality of the diet was improved by higher consumption of fruit, vegetables, fiber and protein, compared to normal intake [57].

Antioxidants reduce oxidative stress and may prevent later complications. Bahadoran et al. proved that a diet rich in antioxidant nutrients (vitamin C, E, β-carotene) improves glucose metabolism and plays a significant role in the prevention of CVD [58]. It was observed that higher calcium intake was significantly associated with lower MetS occurrence, improved BP and increased insulin sensitivity [59,60]. Bian et al. conducted a study demonstrating that consumption of foods rich in B vitamins negatively correlated with the risk of MetS [61].

#### 4.2.2. Lifestyle and Physical Activity

Research suggests that the quality and duration of sleep may have a positive effect on the management of childhood obesity by reducing food consumption, which contributes to body weight loss [62]. In addition, inadequate duration of sleep is associated with a decrease in insulin sensitivity in patients with T1DM [63].

WHO recommends that moderate physical activity among children and adolescents should last at least 60 min a day [64]. In people with DM, it is particularly important because it improves insulin sensitivity [65]. Therefore, it is crucial to include exercise in a child’s routine, e.g.: through family walks, using a pedometer to record the number of steps taken, so that the child can check his achievements on an ongoing basis and share them with friends, which can increase motivation for a more active lifestyle. Parents should encourage their children to attend school sport clubs [66]. A systematic review by Quirk et al. shows an association of physical activity with a statistically significant decrease in some MetS components, such as level of triglycerides and total cholesterol. There were no significant differences in the concentrations of HDL and LDL cholesterol [67]. Salem et al. conducted a study among adolescents with T1DM and observed significantly decreased HbA1c values, insulin requirements, BMI and waist circumference in exercise groups (1 and 3 times a week) [68].

#### 4.2.3. Combined Approach

The most effective intervention to reduce or treat MetS is a combined approach, involving control energy and diet quality along with increasing energy requirements through physical activity. To achieve this, dietary counseling is necessary to help the parents of young patients. The above approach is likely to result in a noticeable decrease in MetS over time, as was the case in the study by Caranti et al., which reports a reduction from the initial 27% of children with MetS to 8.3% after one year of using the combined intervention [69,70].

There are few research papers on the long-term benefits of lifestyle interventions. One of the key approaches is to strike an energy balance between consumption of calories and energy expenditure with an appropriate insulin therapy aimed at avoiding hypoglycemic episodes. This is possible to achieve with the support of a multidisciplinary team with a dietitian involved. Due to insufficient evidence, the Adult Diabetes Prevention Program can serve as an example [71]. It has been confirmed that intensive lifestyle change is associated with a reduction in the severity of MetS and diabetic complications. It should be applied to pediatric diabetic populations in which such an intervention could improve metabolic management and achieve long-term pro-health effects. However, at the beginning of 2021, a protocol in the Cochrane database was developed that presents a reliable source of knowledge on nutritional interventions linked to physical activity in the form of a systematic review and meta-analysis [72].

#### 4.2.4. Gut Microflora

Current research describes the crucial role of the gut microflora in the pathogenesis of both major types of DM [73]. Increased numbers of *Bacteroides* and *Streptococci* and decreased levels of *Clostridium IV* and *XIVa* clusters may contribute to T1DM progression, possibly causing inflammation. Knip and Siljander highlight that the role of the microflora may be important in preventing the onset and aggravation of the T1DM process if the type of healthy gut microflora could be established at birth [74]. Even though chronic low-grade inflammation is not a defining criterion for MetS, it is a proven factor in the etiopathogenesis of obesity, but also insulin resistance, and thus is closely related to the metabolic disturbances in MetS. The role of intestinal permeability in chronic low-grade inflammation confirms the importance of the microbiome, especially in the case of metabolic disorders [75]. Thaiss et al. conducted a study that demonstrated a relationship between glycemia, inflammation and intestinal permeability in both animal and human models. By inducing intestinal hyperglycemia, the authors discovered that HbA1c was a marker positively connected with an increase in serum receptor pathogen recognition (PRR) ligands [76]. This may imply that the MetS criterion should also include the relationship between impaired metabolic control, inflammation and the gut microbiome, which can be modified through eating habits and lifestyle [75]. Most studies report differences in the composition of the gut microbiota between lean and obese subjects (increase in *Firmicutes* and decrease in *Bacteroidetes*) [77,78]. Several studies have confirmed that there is a difference between the microbiota in people with T1DM and healthy ones [79,80]. The microflora of DM patients has a pro-inflammatory phenotype. So far, the causes have not been identified, but research results indicate that the duodenum should be considered a therapeutic target for the inflammatory processes that occur in autoimmune diseases. Increased intestinal permeability is regarded as a potential mediator of the occurrence of T1DM and may be altered by restoring the normal microbiome [81]. The consumed food, and more precisely its nutrients such as sugars, fiber, resistant starch, fats and proteins, affects the type of gut microbiome. Artificial sweeteners are among the ingredients that can negatively influence not only the microbiota but also blood glucose levels [82]. It has been observed in people with known insulin resistance that the plasma metabolome is high in branched-chain amino acids (BCAAs) and in the gut microbes that synthesize them [83]. This may be an important issue for people with newly diagnosed T1DM because a significant relationship has been demonstrated between high consumption of BCAAs and omega-3 fatty acids and the maintenance of β cell function [84].

#### 4.2.5. Pharmacological Support

There is little evidence for pharmacological support, mainly small studies on the use of metformin and dapagliflozin. In the case of the former drug, according to a systematic review by Al Khalifah et al., a small but statistically significant decrease in body weight (−1.46 kg; *p* < 0.01), BMI Z-score by 0.1 (*p* < 0.05), and insulin requirements (−0.16 units/kg/day; *p* < 0.0001) have been observed. There were no significant changes in HbA1c [85]. As for dapagliflozin, which is an inhibitor of sodium-glucose co-transporter 2 (SGLT2-I), one conference report examined its effect at a dose of 10 mg/day for 12 months in three girls with T1DM aged 15 ± 2 years. After 6 months, there was a reduction in the insulin dose (from 58 ± 16 to 35 ± 7 U/day), glucose level (from 191 ± 24 to 171 ± 34 mg/dL), and BMI (from 1.42 ± 0.7 to 0.75 ± 0.8 SDS), but HbA1c did not change. After another 6 months of observation, the above parameters abruptly rose in spite of the initial drop (51 ± 6 U/day; 177 ± 22 mg/dL; 0.86 SDS, respectively). It was also reported that the side effects of the drugs included euglycemic ketosis and hand tremor, and the need for randomized controlled trials was emphasized [86]. In the group of adults with T1DM, a randomized double-blind study was conducted with a dose of 5 and 10 mg/day. After 24 weeks of research, positive results were obtained in the form of an improvement in HbA1c results and weight loss without an increased number of hypoglycemic episodes [87]. However, further testing of this drug is still required, especially in the pediatric group.

#### 4.2.6. Bariatric Surgery

Bariatric surgery, undertaken only in specific cases (especially severe obesity), is one of the most invasive types of treatment for MetS. A study was conducted on a group of 13 obese T1DM patients with average age of 39 years, who had undergone surgery using gastric bypass (*n* = 6) and sleeve gastrectomy (*n* = 7). Comparable benefits were demonstrated in all comparator groups (T1DM, T2DM, control). After 12 months, the median HbA1c decreased (8.3% vs. 7.6%), and the mean insulin dose was reduced (0.8 vs. 0.45 U/kg/day) [88]. Data from scientific publications discussing the use of bariatric surgery concern mainly teenagers. Inge et al. investigated the changes occurring within 5 years after surgery in a larger group, including 161 adolescents. It was observed that 60% of participants maintained weight loss of above 20%. At the beginning, T2DM was present in 14% patients, and almost 90% were taking medications for DM; a year after the surgery, these percentages decreased, and 4 years later, DM was observed only in 2% and none of the people was taking any medications anymore. Furthermore, there were reductions in the incidence of arterial hypertension (30% vs. 15%) and the percentage of people taking antihypertensive drugs (57% vs. 11%). The proportion of patients with hypertriglyceridemia and low HDL cholesterol (36% vs. 6%; 53% vs. 13%) was lower. Death occurred in 3 adolescents (one—3 years after surgery probably from sepsis after a hypoglycemic episode, and two—4 years after surgery due to drug overdose) [89]. However, this method should be a definitive approach, considered and supported by rational arguments and only as a last resort.

#### 4.2.7. Strength and Limitations

The strength of this systematic review is that it identifies gaps in the literature regarding the increasing prevalence of MetS even in children with T1DM. The results of this review have revealed that there is a need for further research in this area. Many studies assess the prevalence of overweight and obesity as well as various blood parameters of people with T1DM, especially in the adult population. However, only in a few studies they are all analyzed together and considered in terms of MetS.

There are also some limitations that should be mentioned. Despite the great efforts of the authors to perform a reliable manual search of databases and literature reference lists, it is possible that some studies that could be included in the review were omitted. Additionally, the fact that this work consists of cross-sectional studies may potentially cause a risk of bias, due to the small number of publications that met the inclusion criteria. In addition, most of the included studies were carried out on populations of European and Middle Eastern descent, which may have an impact on the range of results, due to the different national standards set in the countries of study.

The consequences of obesity, MetS and T1DM are closely related so it is sometimes difficult to distinguish between them. Excessive body weight significantly affects the course of DM, resulting in deterioration of insulin sensitivity and metabolic control. Entering adult life with such a burden may mean increased CVD risk and faster development of diabetic complications.

## 5. Conclusions

The above review indicates that currently one of the important issues is not only the increasing percentage of overweight and obese children and adolescents with T1DM, but also the new additional problem of increased MetS incidence. Research is required to investigate this problem in more depth and on much larger diabetic populations. Analysis of relevant studies may be helpful in developing new guidelines that are effective in reducing the occurrence of MetS among children with T1DM, who may face a range of complications in adulthood.

## Figures and Tables

**Table 1:** Definitions of metabolic syndrome (MetS) in children.

	IDF			ATP	WHO	Weiss et al.
Age group (years)	<10	10–16	>16	-	-	-
Criteria	Abdominal obesity + two or more of the four criteria			Any three of the five criteria	GI * + two or more of the other components	Any three of the five criteria
Abdominal obesity	≥90th percentile WC		WC: ≥94 cm for Europid men, ≥80 cm for Europid women, with ethnicity specific values for other groups	≥90th WC percentile	WHR >0.9 in males, >0.85 in females or/and BMI>30 kg/m^2^	BMI z-score ≥2
Triglycerides	**	≥1.7 mmol/L (≥150 mg/dL)				≥95th percentile
HDL- cholesterol	**	<1.03 mmol/L (<40 mg/dL)	<1.03 mmol/L (<40 mg/dL) in men <1.29 mmol/L (<50 mg/dL) in women or treatment for lipid abnormalities		<0.91 mmol/L (<35 mg/dL) in men <1.01 mmol/L (<39 mg/dL) in women	≤5th percentile
Blood pressure	**	Systolic ≥ 130 / diastolic ≥ 85 mmHg or treatment hypertension		≥90th percentile (age-, sex- and race-specific)	Systolic ≥140 Diastolic ≥90 mmHg	≥95th percentile
Fasting glucose levels	**	≥5.6 mmol/L (100 mg/dL) or known diabetes mellitus			≥6.1 mmol/L (110 mg/dL), which has been changes to ≥ 5.6 mmol/L (100 mg/dL) *	GI (ADA criteria)
Microalbuminuria	-				Urinary albumin excretion rate ≥20 µg/min or albumin/creatinine ratio ≥30 mg/g	-

Abbreviations: American Diabetes Association (ADA), body mass index (BMI), glucose intolerance (GI), high-density lipoprotein (HDL), International Diabetes Federation (IDF), National Cholesterol Education Program Adult Treatment Panel III (ATP), World Health Organization (WHO), waist circumference (WC), waist-hip ratio (WHR); * Or impaired glucose regulation or diabetes mellitus and/or insulin resistance; ** MetS cannot be diagnosed unless there are disturbances in these parameters in the family history.

**Table 2:** Data extraction format for studies included to compute the prevalence of MetS in children with T1DM.

Author	Country	Sample Size Total (F/M)	Age (Year)	Duration of T1DM (Years)	HbA1c (%)	Over- Weight	Obesity	Diagnosis of MetS		Components of MetS			
								Total	F/M	Ab. Obesity	Low HDL	High TG	High BP
		*n*	Min–Max	$\bar{x}$ ± SD Me (IQR)	$\bar{x}$ ± SD Me (IQR)	*n* (%)		*n* (%)	*n* (%)	*n* (%)	*n* (%)	*n* (%)	*n* (%)
Castro-Correia [14]	Portugal	42	14–18	9.2±3.6	8.6±1	18		n/d	n/d	17 (40.5%)	7 (16.7%)	4 (9.5%)	7 (16.7%)
		(42/-)				(42.9%)							
Köken [15]	Turkey	200	8–18	4.6 ± 3.3	8.4 ± 1.6	19	17	17 (8.5%) ^WHO^	11 (11.5%)	n/d	n/d	n/d	24 (12.0%)
		(96/104)				(9.5%)	(8.5%)	21 (10.5%) ^IDF^	/10 (9.6%)				
								27 (13.5%) ^ATP^					
Łuczyński [16]	Poland	500	4–18	4.4 (2.1–7.0)	n/a	78 (15.6%)	73 (14.6%)	16 (3.2%) ^IDF^	n/d	67 (13.4%)	32 (6.4%)	31 (6.2%)	24 (4.8%)
		(245/255)											
Saki [17]	Iran	87	4–21	8.0 ± 3.9	8 ± 3	n/d	n/d	21 (24.1%) ^IDF^	11 (23.2%) /10 (24.8%)	6 (6.0%)	32 (36.8%)	32 (36.8%)	13 (14.9%)
		(48/39)											
Saki [18]	Iran	87	4–21	8.0 ± 3.9	8 ± 3	n/d	n/d	26 (29.9%) ^ATP^	14 (29.2%) /12 (30.7%)	1 (1.1%)	33 (41.7%)	48 (55.2%)	13 (14.9%)
		(48/39)											
Soliman [19]	Egypt	160	<18	5.7 ± 3	9 ± 2.2	n/d	n/d	21 (13.1%) ^IDF^	15 (18.1%) /6 (7.8%)	n/d	n/d	n/d	n/d
		(83/77)											
Szadkowska [20]	Poland	163	10–18	6.2 ± 4.2	8 ± 1.5	n/d	n/d	14 (8.6%) ^IDF^	8 (11.1%) /6 (6.6%)	32 (19.6%)	4 (2.5%)	14 (8.6%)	33 (20.3%)
		(72/91)											
Van Vliet [21]	The Netherlands	283	3–18	5.3 (2.9–8.6)	8.3 (7.5–9.8)	83 (29.3%)	26 (9.2%)	81 (28.6%) ^WEISS^	n/d	26 (9.2%)	60 (21.2%)	49 (17.3%)	37 (13.1%)
		(145/138)											
Valerio [22]	Italy	412	16–19	8.4 ± 3.9	8.9 ± 1.7	101 (24.5%)	16 (3.9%)	39 (9.5%) ^IDF^	31 (16.1%) /8 (3.7%)	83 (20.1%)	66 (16.0%)	23 (5.6%)	73 (17.7%)
		(193/219)											

Abbreviations: abdominal (Ab), National Cholesterol Education Program Adult Treatment Panel III (ATP), blood pressure (BP), females (F), high-density lipoprotein (HDL), International Diabetes Federation (IDF), interquartile range (IQR), males (M), median (Me), no data (n/d), number of participants (*n*), metabolic syndrome (MetS), standard deviation (SD), type 1 diabetes mellitus (T1DM), triglycerides (TG), World Health Organization (WHO), mean ($\bar{x})$.

## Data Availability

No new data were created or analyzed in this study. Data sharing is not applicable to this article.

## Supplementary Materials

The following are available online at https://www.mdpi.com/article/10.3390/nu13061782/s1, Table S1. MeSH terms; Table S2. The assessment of methodological quality included studies.

## References

[1] American Diabetes Association (2021). Classification and Diagnosis of Diabetes: Standards of Medical Care in Diabetes—2021. Diabetes Care.

[2] International Diabetes Federation (2019). IDF Diabetes Atlas. https://www.diabetesatlas.org.

[3] Minges K.E., Whittemore R., Grey M. (2013). Overweight and obesity in youth with type 1 diabetes. Annu. Rev. Nurs. Res..

[4] Purnell J.Q., Zinman B., Brunzell J.D. (2013). The effect of excess weight gain with intensive diabetes mellitus treatment on cardiovascular disease risk factors and atherosclerosis in type 1 diabetes mellitus: Results from the Diabetes Control and Complications Trial/Epidemiology of Diabetes Interventions and Complications Study (DCCT/EDIC) study. Circulation.

[5] Alberti K.G., Zimmet P., Shaw J. (2006). Metabolic syndrome—A new world-wide definition. A Consensus Statement from the International Diabetes Federation. Diabet. Med..

[6] Zimmet P., Alberti K.G.M., Kaufman F., Tajima N., Silink M., Arslanian S., Wong G., Bennett P., Shaw J., Caprio S. (2007). The metabolic syndrome in children and adolescents—An IDF consensus report. Pediatr. Diabetes.

[7] Steinberger J., Daniels S.R., Eckel R.H., Hayman L., Lustig R.H., McCrindle B., Mietus-Snyder M.L. (2009). Progress and Challenges in Metabolic Syndrome in Children and Adolescents. Circulation.

[8] Davis T.M., Bruce D.G., Davis W.A. (2007). Prevalence and prognostic implications of the metabolic syndrome in community-based patients with type 1 diabetes: The Fremantle Diabetes Study. Diabetes Res. Clin. Pract..

[9] Liberati A., Altman D.G., Tetzlaff J., Mulrow C., Gøtzsche P.C., Ioannidis J.P., Clarke M., Devereaux P.J., Kleijnen J., Moher D. (2009). The PRISMA statement for reporting systematic reviews and meta-analyses of studies that evaluate health care interventions: Explanation and elaboration. PLoS Med..

[10] Expert Panel on Detection, Evaluation, and Treatment of High Blood Cholesterol in Adults (2001). Executive Summary of The Third Report of The National Cholesterol Education Program (NCEP). JAMA.

[11] Weiss R., Dziura J., Burgert T.S., Tamborlane W.V., Taksali S.E., Yeckel C.W., Allen K., Lopes M., Savoye M., Morrison J. (2004). Obesity and the Metabolic Syndrome in Children and Adolescents. N. Engl. J. Med..

[12] World Health Organization (1999). Definition, Diagnosis and Classification of Diabetes Mellitus and Its Complications: Report of A WHO Consultation. Part 1, Diagnosis and Classification of Diabetes Mellitus.

[13] National Institutes of Health Quality Assessment Tool for Observational Cohort and Cross-Sectional Studies. https://www.nhlbi.nih.gov/health-topics/study-quality-assessment-tools.

[14] Castro-Correia C., Santos-Silva R., Pinheiro M., Costa C., Fontoura M. (2018). Metabolic risk factors in adolescent girls with type 1 diabetes. J. Pediatr. Endocrinol. Metab..

[15] Yayıcı Köken Ö., Kara C., Can Yılmaz G., Aydın H.M. (2020). Prevalence of Obesity and Metabolic Syndrome in Children with Type 1 Diabetes: A Comparative Assessment Based on Criteria Established by the International Diabetes Federation, World Health Organisation and National Cholesterol Education Program. J. Clin. Res. Pediatr. Endocrinol..

[16] Łuczyński W., Szypowska A., Głowińska-Olszewska B., Bossowski A. (2011). Overweight, obesity and features of metabolic syndrome in children with diabetes treated with insulin pump therapy. Eur. J. Pediatr..

[17] Saki F. (2016). Prevalence of Metabolic Syndrome in Children with Type 1 Diabetes in South of Iran. J. Compr. Pediatr..

[18] Saki F., Setoodehnia Z., Javanmardi H., Omrani G. (2016). Association between Metabolic Syndrome Criteria and Body-composition Components in Children with Type 1 Diabetes Mellitus. Int. J. Pediatr.

[19] Soliman H.M., Mosaad Y.O., Ibrahim A. (2019). The prevalence and the clinical profile of metabolic syndrome in children and adolescents with Type 1 diabetes. Diabetes Metab. Syndr..

[20] Szadkowska A., Pietrzak I., Szlawska J., Kozera A., Gadzicka A., Młynarski W. (2009). Abdominal obesity, metabolic syndrome in type 1 diabetic children and adolescents. Pediatr. Endocrinol. Diabetes Metab..

[21] Van Vliet M., Van der Heyden J.C., Diamant M., Von Rosenstiel I.A., Schindhelm R.K., Aanstoot H.J., Veeze H.J. (2010). Overweight Is Highly Prevalent in Children with Type 1 Diabetes And Associates with Cardiometabolic Risk. J. Pediatr..

[22] Valerio G., Iafusco D., Zucchini S., Maffeis C. (2012). Abdominal adiposity and cardiovascular risk factors in adolescents with type 1 diabetes. Diabetes Res. Clin. Pract..

[23] Maffeis C., Birkebaek N.H., Konstantinova M., Schwandt A., Vazeou A., Casteels K., Jali S., Limbert C., Pundziute-Lycka A., Toth-Heyn P. (2018). Prevalence of underweight, overweight, and obesity in children and adolescents with type 1 diabetes: Data from the international SWEET registry. Pediatr. Diabetes.

[24] NCD Risk Factor Collaboration (2017). Worldwide trends in body-mass index, underweight, overweight, and obesity from 1975 to 2016: A pooled analysis of 2416 population-based measurement studies in 128·9 million children, adolescents, and adults. Lancet.

[25] De Keukelaere M., Fieuws S., Reynaert N., Vandoorne E., Kerckhove K.V., Asscherickx W., Casteels K. (2018). Evolution of body mass index in children with type 1 diabetes mellitus. Eur. J. Pediatr..

[26] Kaminsky L.A., Dewey D. (2014). The association between body mass index and physical activity, and body image, self esteem and social support in adolescents with type 1 diabetes. Can. J. Diabetes.

[27] Friend A., Craig L., Turner S. (2013). The prevalence of metabolic syndrome in children: A systematic review of the literature. Metab. Syndr. Relat. Disord..

[28] Bonadonna R., Cucinotta D., Fedele D., Riccardi G., Tiengo A. (2006). The metabolic syndrome is a risk indicator of microvascular and macrovascular complications in diabetes: Results from Metascreen, a multicenter diabetes clinic-based survey. Diabetes Care.

[29] Kułaga Z., Litwin M., Tkaczyk M., Palczewska I., Zajączkowska M., Zwolińska D., Krynicki T., Wasilewska A., Moczulska A., Morawiec-Knysak A. (2011). Polish 2010 growth references for school-aged children and adolescents. Eur. J. Pediatr..

[30] Kułaga Z., Litwin M., Grajda A., Kułaga K., Gurzkowska B., Góźdź M., Pan H. (2012). Oscillometric blood pressure percentiles for Polish normal-weight school-aged children and adolescents. J. Hypertens..

[31] Ahrens W., Moreno L.A., Mårild S., Molnár D., Siani A., De Henauw S., Böhmann J., Günther K., Hadjigeorgiou C., Iacoviello L. (2014). Metabolic syndrome in young children: Definitions and results of the IDEFICS study. Int. J. Obes..

[32] Ferranti S.D.d., Gauvreau K., Ludwig D.S., Neufeld E.J., Newburger J.W., Rifai N. (2004). Prevalence of the Metabolic Syndrome in American Adolescents. Circulation.

[33] Chillarón J.J., Flores-Le-Roux J.A., Goday A., Benaiges D., Carrera M.J., Puig J., Cano-Pérez J.F., Pedro-Botet J. (2010). Metabolic syndrome and type-1 diabetes mellitus: Prevalence and associated factors. Rev. Esp. Cardiol..

[34] Ghosh S., Collier A., Hair M., Malik I., Elhadd T. (2010). Metabolic syndrome in type 1 diabetes. Int. J. Diabetes Mellit..

[35] McGill M., Molyneaux L., Twigg S.M., Yue D.K. (2008). The metabolic syndrome in type 1 diabetes: Does it exist and does it matter?. J. Diabetes Complicat..

[36] Vestberg D., Rosengren A., Olsson M., Gudbjörnsdottir S., Svensson A.-M., Lind M. (2013). Relationship Between Overweight and Obesity with Hospitalization for Heart Failure in 20,985 Patients With Type 1 Diabetes. Diabetes Care.

[37] Dietz W.H. (1998). Health consequences of obesity in youth: Childhood predictors of adult disease. Pediatrics.

[38] Krishnan S., Short K.R. (2009). Prevalence and significance of cardiometabolic risk factors in children with type 1 diabetes. J. Cardiometab. Syndr..

[39] Pozzilli P., Guglielmi C., Caprio S., Buzzetti R. (2011). Obesity, autoimmunity, and double diabetes in youth. Diabetes Care.

[40] Pérez A., Wägner A.M., Carreras G., Giménez G., Sánchez-Quesada J.L., Rigla M., Gómez-Gerique J.A., Pou J.M., de Leiva A. (2000). Prevalence and phenotypic distribution of dyslipidemia in type 1 diabetes mellitus: Effect of glycemic control. Arch. Intern. Med..

[41] Molitch M.E., Rupp D., Carnethon M. (2006). Higher levels of HDL cholesterol are associated with a decreased likelihood of albuminuria in patients with long-standing type 1 diabetes. Diabetes Care.

[42] Chillarón J.J., Sales M.P., Flores-Le-Roux J.A., Murillo J., Benaiges D., Castells I., Goday A., Cano J.F., Pedro-Botet J. (2011). Insulin resistance and hypertension in patients with type 1 diabetes. J. Diabetes Complicat..

[43] Raitakari O.T., Porkka K.V.K., Räsänen L., Rönnemaa T., Viikari J.S.A. (1994). Clustering and six year cluster-tracking of serum total cholesterol, HDL-cholesterol and diastolic blood pressure in children and young adults The cardiovascular risk in young finns study. J. Clin. Epidemiol..

[44] Pandit D., Chiplonkar S., Khadilkar A., Kinare A., Khadilkar V. (2011). Efficacy of a continuous metabolic syndrome score in Indian children for detecting subclinical atherosclerotic risk. Int. J. Obes..

[45] Styne D.M., Arslanian S.A., Connor E.L., Farooqi I.S., Murad M.H., Silverstein J.H., Yanovski J.A. (2017). Pediatric Obesity-Assessment, Treatment, and Prevention: An Endocrine Society Clinical Practice Guideline. J. Clin. Endocrinol. Metab..

[46] Kamath C.C., Vickers K.S., Ehrlich A., McGovern L., Johnson J., Singhal V., Paulo R., Hettinger A., Erwin P.J., Montori V.M. (2008). Clinical review: Behavioral interventions to prevent childhood obesity: A systematic review and metaanalyses of randomized trials. J. Clin. Endocrinol. Metab..

[47] Overby N.C., Flaaten V., Veierød M.B., Bergstad I., Margeirsdottir H.D., Dahl-Jørgensen K., Andersen L.F. (2007). Children and adolescents with type 1 diabetes eat a more atherosclerosis-prone diet than healthy control subjects. Diabetologia.

[48] Maffeis C., Morandi A., Ventura E., Sabbion A., Contreas G., Tomasselli F., Tommasi M., Fasan I., Costantini S., Pinelli L. (2012). Diet, physical, and biochemical characteristics of children and adolescents with type 1 diabetes: Relationship between dietary fat and glucose control. Pediatr. Diabetes.

[49] Øverby N.C., Margeirsdottir H.D., Brunborg C., Dahl-Jørgensen K., Andersen L.F. (2008). Sweets, snacking habits, and skipping meals in children and adolescents on intensive insulin treatment. Pediatr. Diabetes.

[50] Patton S.R., Dolan L.M., Powers S.W. (2007). Dietary Adherence and Associated Glycemic Control in Families of Young Children with Type 1 Diabetes. J. Am. Diet. Assoc..

[51] Castro-Barquero S., Ruiz-León A.M., Sierra-Pérez M., Estruch R., Casas R. (2020). Dietary Strategies for Metabolic Syndrome: A Comprehensive Review. Nutrients.

[52] DeBoer M.D. (2019). Assessing and Managing the Metabolic Syndrome in Children and Adolescents. Nutrients.

[53] Velázquez-López L., Santiago-Díaz G., Nava-Hernández J., Muñoz-Torres A.V., Medina-Bravo P., Torres-Tamayo M. (2014). Mediterranean-style diet reduces metabolic syndrome components in obese children and adolescents with obesity. BMC Pediatr..

[54] Chan T.F., Lin W.T., Huang H.L., Lee C.Y., Wu P.W., Chiu Y.W., Huang C.C., Tsai S., Lin C.L., Lee C.H. (2014). Consumption of sugar-sweetened beverages is associated with components of the metabolic syndrome in adolescents. Nutrients.

[55] Scharf R.J., DeBoer M.D. (2016). Sugar-Sweetened Beverages and Children’s Health. Annu. Rev. Public Health.

[56] Asghari G., Yuzbashian E., Mirmiran P., Hooshmand F., Najafi R., Azizi F. (2016). Dietary Approaches to Stop Hypertension (DASH) Dietary Pattern Is Associated with Reduced Incidence of Metabolic Syndrome in Children and Adolescents. J. Pediatr..

[57] Peairs A.D., Shah A.S., Summer S., Hess M., Couch S.C. (2017). Effects of the dietary approaches to stop hypertension (DASH) diet on glucose variability in youth with Type 1 diabetes. Diabetes Manag..

[58] Bahadoran Z., Golzarand M., Mirmiran P., Shiva N., Azizi F. (2012). Dietary total antioxidant capacity and the occurrence of metabolic syndrome and its components after a 3-year follow-up in adults: Tehran Lipid and Glucose Study. Nutr. Metab..

[59] Liu S., Song Y., Ford E.S., Manson J.E., Buring J.E., Ridker P.M. (2005). Dietary calcium, vitamin D, and the prevalence of metabolic syndrome in middle-aged and older U.S. women. Diabetes Care.

[60] Zemel M.B. (1998). Nutritional and endocrine modulation of intracellular calcium: Implications in obesity, insulin resistance and hypertension. Mol. Cell Biochem..

[61] Bian S., Gao Y., Zhang M., Wang X., Liu W., Zhang D., Huang G. (2013). Dietary nutrient intake and metabolic syndrome risk in Chinese adults: A case-control study. Nutr. J..

[62] Hart C.N., Hawley N.L., Wing R.R. (2016). Development of a Behavioral Sleep Intervention as a Novel Approach for Pediatric Obesity in School-aged Children. Pediatr. Clin. N. Am..

[63] Donga E., van Dijk M., van Dijk J.G., Biermasz N.R., Lammers G.-J., van Kralingen K., Hoogma R.P.L.M., Corssmit E.P.M., Romijn J.A. (2010). Partial Sleep Restriction Decreases Insulin Sensitivity in Type 1 Diabetes. Diabetes Care.

[64] World Health Organization (2020). WHO Guidelines Approved by the Guidelines Review Committee. WHO Guidelines on Physical Activity and Sedentary Behaviour.

[65] Guinhouya B.C., Samouda H., Zitouni D., Vilhelm C., Hubert H. (2011). Evidence of the influence of physical activity on the metabolic syndrome and/or on insulin resistance in pediatric populations: A systematic review. Int. J. Pediatr. Obes..

[66] Nader P.R., Bradley R.H., Houts R.M., McRitchie S.L., O’Brien M. (2008). Moderate-to-vigorous physical activity from ages 9 to 15 years. JAMA.

[67] Quirk H., Blake H., Tennyson R., Randell T.L., Glazebrook C. (2014). Physical activity interventions in children and young people with Type 1 diabetes mellitus: A systematic review with meta-analysis. Diabet. Med..

[68] Salem M.A., AboElAsrar M.A., Elbarbary N.S., ElHilaly R.A., Refaat Y.M. (2010). Is exercise a therapeutic tool for improvement of cardiovascular risk factors in adolescents with type 1 diabetes mellitus? A randomised controlled trial. Diabetol. Metab. Syndr..

[69] Caranti D.A., de Mello M.T., Prado W.L., Tock L., Siqueira K.O., de Piano A., Lofrano M.C., Cristofalo D.M.J., Lederman H., Tufik S. (2007). Short- and long-term beneficial effects of a multidisciplinary therapy for the control of metabolic syndrome in obese adolescents. Metabolism.

[70] Leite N., Milano G., Cieslak F., Lopes W., Rodacki A., Radominski R. (2009). Effects of physical exercise and nutritional guidance on metabolic syndrome in obese adolescents. Braz. J. Phys. Ther..

[71] DeBoer M.D., Filipp S.L., Gurka M.J. (2018). Use of a Metabolic Syndrome Severity Z Score to Track Risk During Treatment of Prediabetes: An Analysis of the Diabetes Prevention Program. Diabetes Care.

[72] Shorey S., Ng E., Law E.C., Wong J.C.M., Loke K., Tam W.W. (2021). Physical activity interventions and nutrition-based interventions for children and adolescents with type 1 diabetes mellitus. Cochrane Cochrane Database Syst. Rev..

[73] Blandino G., Inturri R., Lazzara F., Di Rosa M., Malaguarnera L. (2016). Impact of gut microbiota on diabetes mellitus. Diabetes Metab..

[74] Knip M., Siljander H. (2016). The role of the intestinal microbiota in type 1 diabetes mellitus. Nat. Rev. Endocrinol..

[75] Dabke K., Hendrick G., Devkota S. (2019). The gut microbiome and metabolic syndrome. J. Clin. Investig..

[76] Thaiss C.A., Levy M., Grosheva I., Zheng D., Soffer E., Blacher E., Braverman S., Tengeler A.C., Barak O., Elazar M. (2018). Hyperglycemia drives intestinal barrier dysfunction and risk for enteric infection. Science.

[77] Ley R.E., Bäckhed F., Turnbaugh P., Lozupone C.A., Knight R.D., Gordon J.I. (2005). Obesity alters gut microbial ecology. Proc. Natl. Acad. Sci. USA.

[78] Turnbaugh P.J., Ley R.E., Mahowald M.A., Magrini V., Mardis E.R., Gordon J.I. (2006). An obesity-associated gut microbiome with increased capacity for energy harvest. Nature.

[79] Soyucen E., Gulcan A., Aktuglu-Zeybek A.C., Onal H., Kiykim E., Aydin A. (2014). Differences in the gut microbiota of healthy children and those with type 1 diabetes. Pediatr. Int..

[80] Murri M., Leiva I., Gomez-Zumaquero J.M., Tinahones F.J., Cardona F., Soriguer F., Queipo-Ortuño M.I. (2013). Gut microbiota in children with type 1 diabetes differs from that in healthy children: A case-control study. BMC Med..

[81] Pellegrini S., Sordi V., Bolla A.M., Saita D., Ferrarese R., Canducci F., Clementi M., Invernizzi F., Mariani A., Bonfanti R. (2017). Duodenal Mucosa of Patients with Type 1 Diabetes Shows Distinctive Inflammatory Profile and Microbiota. J. Clin. Endocrinol. Metab..

[82] Suez J., Korem T., Zeevi D., Zilberman-Schapira G., Thaiss C.A., Maza O., Israeli D., Zmora N., Gilad S., Weinberger A. (2014). Artificial sweeteners induce glucose intolerance by altering the gut microbiota. Nature.

[83] Pedersen H.K., Gudmundsdottir V., Nielsen H.B., Hyotylainen T., Nielsen T., Jensen B.A., Forslund K., Hildebrand F., Prifti E., Falony G. (2016). Human gut microbes impact host serum metabolome and insulin sensitivity. Nature.

[84] Mayer-Davis E.J., Dabelea D., Crandell J.L., Crume T., D’Agostino R.B., Dolan L., King I.B., Lawrence J.M., Norris J.M., Pihoker C. (2013). Nutritional factors and preservation of C-peptide in youth with recently diagnosed type 1 diabetes: SEARCH Nutrition Ancillary Study. Diabetes Care.

[85] Al Khalifah R.A., Alnhdi A., Alghar H., Alanazi M., Florez I.D. (2017). The effect of adding metformin to insulin therapy for type 1 diabetes mellitus children: A systematic review and meta-analysis. Pediatr Diabetes.

[86] Roman R., Valdivia N., Ruiz S. (2016). Overweight adolescents with type 1 diabetes may decrease body mass index, insulin dose and glucose variability on dapagliflozin, a SGLT2 inhibitor. Horm. Res. Paediatr..

[87] Dandona P., Mathieu C., Phillip M., Hansen L., Griffen S.C., Tschöpe D., Thorén F., Xu J., Langkilde A.M., Proietto J. (2017). Efficacy and safety of dapagliflozin in patients with inadequately controlled type 1 diabetes (DEPICT-1): 24 week results from a multicentre, double-blind, phase 3, randomised controlled trial. Lancet Diabetes Endocrinol..

[88] Faucher P., Poitou C., Carette C., Tezenas du Montcel S., Barsamian C., Touati E., Bouillot J.L., Torcivia A., Czernichow S., Oppert J.M. (2016). Bariatric Surgery in Obese Patients with Type 1 Diabetes: Effects on Weight Loss and Metabolic Control. Obes. Surg..

[89] Inge T.H., Courcoulas A.P., Jenkins T.M., Michalsky M.P., Brandt M.L., Xanthakos S.A., Dixon J.B., Harmon C.M., Chen M.K., Xie C. (2019). Five-Year Outcomes of Gastric Bypass in Adolescents as Compared with Adults. N. Engl. J. Med..

