# Supplementary material for: Prevalence of Metabolic Syndrome in Children and Adolescents with Type 1 Diabetes Mellitus and Possibilities of Prevention and Treatment: A Systematic Review
Source: Nutrients. 2021 May 23;13(6):1782. doi: 10.3390/nu13061782 (PMC8224679; doi:10.3390/nu13061782)
Supplement: Supplementary file 1 [file nutrients-13-01782-s001.zip › nutrients-1206241-supplementary.pdf]

Supplementary Material

Prevalence of Metabolic Syndrome in Children and Adolescents with Type 1 Diabetes Mellitus and Possibilities of Prevention and Treatment: A Systematic Review

Monika Grabia , Renata Markiewicz-Żukowska* and Katarzyna Socha

[1] Department of Bromatology, Faculty of Pharmacy with the Division of Laboratory Medicine, Medical University of Białystok, Mickiewicza 2D Street, 15-222 Białystok, Poland; monika.grabia@umb.edu.pl (M.G.); katarzyna.socha@umb.edu.pl (K.S.)
* Correspondence: renmar@poczta.onet.pl; Tel.: +48-85-748-5469

Table S1 MeSH terms.

Search: **(((((((diabetes type 1) OR (T1DM)) OR (T1D)) AND (children)) OR (adolescents)) OR (youth)) OR (teenagers)) AND (metabolic syndrome)**   ((("diabetes mellitus, type 1"[MeSH Terms] OR "type 1 diabetes mellitus"[All Fields] OR "diabetes type 1"[All Fields] OR "T1DM"[All Fields] OR "T1D"[All Fields]) AND ("child"[MeSH Terms] OR "child"[All Fields] OR "children"[All Fields] OR "child s"[All Fields] OR "children s"[All Fields] OR "childrens"[All Fields] OR "childs"[All Fields])) OR ("adolescences"[All Fields] OR "adolescency"[All Fields] OR "adolescent"[MeSH Terms] OR "adolescent"[All Fields] OR "adolescence"[All Fields] OR "adolescents"[All Fields] OR "adolescent s"[All Fields]) OR ("adolescent"[MeSH Terms] OR "adolescent"[All Fields] OR "youth"[All Fields] OR "youths"[All Fields] OR "youth s"[All Fields]) OR ("adolescent"[MeSH Terms] OR "adolescent"[All Fields] OR "teenage"[All Fields] OR "teenager"[All Fields] OR "teenagers"[All Fields] OR "teenaged"[All Fields] OR "teenager s"[All Fields] OR "teenages"[All Fields])) AND ("metabolic syndrome"[MeSH Terms] OR ("metabolic"[All Fields] AND "syndrome"[All Fields]) OR "metabolic syndrome"[All Fields])
**Translations**
**diabetes type 1:** "diabetes mellitus, type 1"[MeSH Terms] OR "type 1 diabetes mellitus"[All Fields] OR "diabetes type 1"[All Fields]
**children:** "child"[MeSH Terms] OR "child"[All Fields] OR "children"[All Fields] OR "child's"[All Fields] OR "children's"[All Fields] OR "childrens"[All Fields] OR "childs"[All Fields]
**adolescents:** "adolescences"[All Fields] OR "adolescency"[All Fields] OR "adolescent"[MeSH Terms] OR "adolescent"[All Fields] OR "adolescence"[All Fields] OR "adolescents"[All Fields] OR "adolescent's"[All Fields]
**youth:** "adolescent"[MeSH Terms] OR "adolescent"[All Fields] OR "youth"[All Fields] OR "youths"[All Fields] OR "youth's"[All Fields]
**teenagers:** "adolescent"[MeSH Terms] OR "adolescent"[All Fields] OR "teenage"[All Fields] OR "teenager"[All Fields] OR "teenagers"[All Fields] OR "teenaged"[All Fields] OR "teenager's"[All Fields] OR "teenages"[All Fields]
**metabolic syndrome:** "metabolic syndrome"[MeSH Terms] OR ("metabolic"[All Fields] AND "syndrome"[All Fields]) OR "metabolic syndrome"[All Fields]

Table S2 The assessment of methodological quality included studies.

Question number	Criteria	Castro-Correia [14]	Köken [15]	Łuczyński [16]	Saki [17]	Saki [18]	Soliman [19]	Szadkowska [20]	Valerio [21]	Van Vliet [22]
1.	Research question	yes	yes	yes	yes	yes	yes	yes	yes	yes
2.	Study population	yes	c/d	yes	yes	yes	yes	yes	yes	yes
3.	Participation rate	no	n/r	n/r	n/r	n/r	n/r	n/r	yes	n/r
4.	Groups recruited from the same population and uniform eligibility criteria	yes	yes	yes	yes	yes	yes	yes	yes	yes
5.	Sample size justification	no	no	no	yes	yes	no	no	no	no
6.	Exposure assessed prior to outcome measurement	no	no	no	no	no	no	no	no	no
7.	Sufficient timeframe of effect	no	no	no	no	no	no	no	no	no
8.	Different levels of the exposure of interest	n/a	n/a	n/a	n/a	n/a	n/a	n/a	n/a	n/a
9.	Exposure measures and assessment	yes	yes	yes	yes	yes	yes	yes	yes	yes
10.	Repeated exposure assessment	no	no	no	no	no	no	no	no	no
11.	Outcome measures	yes	yes	yes	yes	yes	yes	yes	yes	yes
12.	Blinding	n/a	n/a	n/a	n/a	n/a	n/a	n/a	n/a	n/a
13.	Follow-up rate	n/a	n/a	n/a	n/a	n/a	n/a	n/a	n/a	n/a
14.	Confounding	n/r	n/r	yes	n/r	n/r	no	no	yes	yes
	**Quality category**	**fair**	**fair**	**good**	**good**	**good**	**fair**	**fair**	**good**	**good**

Each question could be answered with yes (the item scored 1 points), no or other (c/d, cannot determine; n/a, not applicable; n/r, not reported.
